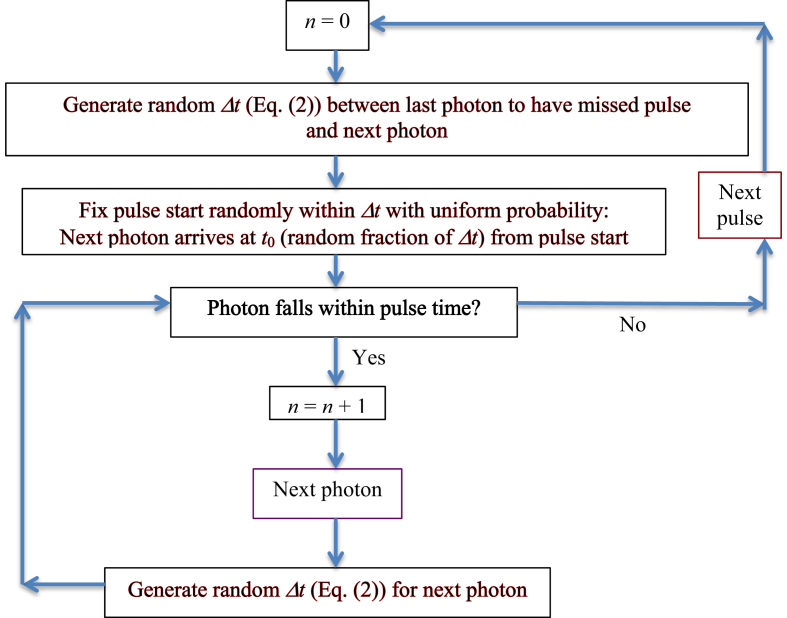# Photon statistics and pulsed-light regime in experiments on unicellular algae

**DOI:** 10.1016/j.heliyon.2022.e12474

**Published:** 2022-12-21

**Authors:** Yair Zarmi

**Affiliations:** a Jacob Blaustein Institutes for Desert Research, Israel; b Ben-Gurion University of the Negev, Israel; c Midreshet Ben-Gurion, 8499000, Israel

**Keywords:** Unicellular algae, Pulsed-light regime, Photon arrival time statistics

## Abstract

Photon arrival time statistics significantly affect the performance of the photosynthetic system under a pulsed-light regime when the average number of photons hitting the Chlorophyll antenna of PS II is small. As a result, the interpretation of measurements of the production rate of biomass or other products of unicellular algae under a pulsed-light regime and the comparison of the results with those obtained under continuous illumination depends crucially upon accounting for these statistics.

## 1 Introduction – photon arrival time statistics

In pulsed-light experiments on unicellular algae, it is customary to compare the long-time average production rate of biomass or other products under a pulsed-light regime with the production rate under continuous illumination at the “integrated” photon flux, defined as:

(1) $$I_{av}=I_{0}\cdot \left(\text{duty}\;\text{cycle}\right)=I_{0}\;\frac{T_{pulse}}{T_{cycle}}\;,(T_{cycle}=T_{pulse}+T_{dark}),$$

where *I*_0_ is the nominal photon flux of the light source during the pulse, *T*_*pulse*_ is the pulse time and *T*_*dark*_ is the dark time. For example, for *I*_0_ = 1,000 *μ*Mol/m^2^/s and a duty cycle of 0.1, *I*_*av*_ = 100 *μ*Mol/m^2^/s.

However, owing to the stochastic nature of photon arrival times, such comparison may be relevant and convey information of significance only when <*n*>, the average number of photons absorbed by the chlorophyll antennae of the photosynthetic apparatus during one pulse is sufficiently large. This requires a combination of a sufficiently long *T*_*pulse*_, associated with the magnitude of the photon absorption cross-section area of the chlorophyll antennae, *A*, in particular, of PS II.

For common light sources, photon statistics is Poissonian (Goodman, 1985; Fox, 2006), specifically, the time gap, *Δt* (in ms), between two consecutive photons hitting the PS II antenna varies randomly under an exponential probability density function:

(2) $$P\left(\Delta t\right)\;d\left(\Delta t\right)=\left(1/\tau \right)\;e^{-\left(\Delta t/\tau \right)}\;d(\Delta t).$$

*τ*, the average time gap is given in ms by:

(3) $$\tau =1/(C\;I_{0}\;A).$$

*I*_0_ is the nominal photon flux in *μ*Mol/m^2^/s, *A* is the photon absorption cross-section area of the PS II chlorophyll antenna (in nm^2^) and *C* = 0.000602215 is the conversion factor of the photon flux from *μ*Mol/m^2^/s to photons/nm^2^/ms. For example, for *I*_0_ = 1,000*μ*Mol/m^2^/s and a chlorophyll antenna with *A* = 1nm^2^, Eq. (3) yields *τ* = 1.66 ms, meaning that, on the average, the antenna absorbs 0.6 photons/ms.

Owing to the randomness of photon arrival times, *n*, the number of photons absorbed by the PS II chlorophyll antenna during a pulse, varies randomly. This randomness affects photosynthetic performance under pulsed light in two ways when <*n*>, the average of *n*, is small. First, the standard deviation of *n* around this average is sizeable. It may be as large as, or greater than, <*n*>. Second, the statistics of the arrival time of the first photon to be absorbed during a pulse affects the results significantly.

The value *A* = 1nm^2^ will be used throughout the paper as a representative value so as to provide an order-of magnitude estimate. The justification is that there are quite a few papers, in which *A* has been estimated to be in the range of a fraction of 1nm^2^ to several nm^2^ (Malkin et al., 1981; Ley, 1984; Greenbaum et al., 1987; Guenther et al., 1988; Mauzerall and Greenbaum, 1989; Falk et al., 1994; Hemelrijk and van Gorkom, 1996; Suggett et al., 2004; de Marchin et al., 2014; Klughammer and Schreiber, 2015; Kim et al., 2020).

Furthermore, as stated several times throughout the paper, what counts is not the separate effect of the photon flux, *I*, and of *A*, the photon absorption cross-section area. Rather, the important entities are <*n*>, the average number of photons absorbed during a pulse, and the statistical fluctuations of the actual number absorbed, *n*, around this average. As shown in Section 2, both depend on (*I A*). Thus, for a given value of <*n*>, one may vary *I* and *A*.

## 2 First-photon arrival time statistics

The concept of “first arrival time” or “first passage time” is studied extensively in the literature on stochastic processes; see, e.g., Stratonovich (1981). Applications range from the planning of bus transportation systems (time scales of minutes to hours) to the observation of photons collected in cosmic radiation (time scales of picoseconds; see, e.g., Murphy, 2001). In all cases, the statistics of the arrival time of the first “signal” (a bus or a photon) affects <*n*>, the average number of signals counted during a finite observation time. If <*n*> is small, the effect may be sizeable.

Photon absorption by the chlorophyll antenna of PS II under a pulsed-light regime is no different. While in commonly used units, (*μ*Mol/m^2^/s), the photons flux is enormous, in the range of 10^20^ − 10^22^ photons/m^2^/s, the corresponding *average* number of photons hitting a chlorophyll antenna with a photon absorption cross-section of 1nm^2^ in one millisecond varies in the range of 0.1–10 photons. For such small numbers, the statistical nature of photon arrival times plays a significant role.

The random photon arrival times are governed by the probability distribution of Eq. (2). Above and beyond the statistical effect described by Eq. (2), the statistical nature of the arrival time of the first photon, which may be absorbed within the pulse time, ALWAYS affects <*n*>. The relative effect is small when <*n*> is large. However, it may be very significant when <*n*> is small: The cycle-averaged light intensity may then be significantly higher than the long-time average (integrated value) of Eq. (1). To see this, consider two extreme scenarios.

Scenario no. 1: The last photon to have missed the pulse arrives exactly at pulse start. The first photon, which may be absorbed within pulse duration, arrives at a time gap, *Δt* (randomly determined by the probability density of Eq. (2)), afterwards.

Scenario no. 2: The first photon is always absorbed exactly at pulse start.

During a pulse of length *T*_*pulse*_, the statistics of Eq. (2) yields:

(4) $${\left\langle n\right\rangle }_{Scenario\;1}=\left(T_{pulse}/\tau \right)\;,{\left\langle n\right\rangle }_{Scenario\;2}=1+(T_{pulse}/\tau ).$$

Given <*n*>, the corresponding cycle-averaged photon flux (in *μ*Mol/m^2^/s) is:

(5) $$I_{av,cycle}=\frac{1}{C}\;\frac{\left\langle n\right\rangle }{A\;T_{cycle}}.$$

Eqs. (4) and (5) yield the following expressions for the cycle-averaged photon flux:

(6) $$I_{av,cycle,Scenario\;1}=I_{0}\cdot \left(\text{duty}\;\text{cycle}\right)\;,I_{av,cycle,Scenario\;2}=\left(\frac{1}{C}\;\frac{1}{A\;T_{pulse}}+I_{0}\right)\cdot (\text{duty}\;\text{cycle}).$$

Thus, using the integrated photon flux (Eq. (1)) is tantamount to adoption of Scenario 1, which does not account for the effect of the first-photon arrival time statistics.

As an example, consider a flux, *I*_0_ = 1,000*μ*Mol/m^2^/s, a chlorophyll antenna of 1nm^2^ (*τ* = 1.66 ms) and a duty cycle of 0.1. For *T*_*pulse*_ = 1 ms and *T*_*dark*_ = 9 ms, Eq. (4) yields:

(7) $${\left\langle n\right\rangle }_{Scenario\;1}=0.602\;photons/pulse\;,{\left\langle n\right\rangle }_{Scenario\;2}=1.602\;photons/pulse.$$

Eq. (5) for the cycle-averaged photon flux of the two scenarios Eq. (6) yields:

(8) $$I_{av,cycle,Scenario\;1}=100\;\mu \text{Mol}/m^{2}/s\;,I_{av,cycle,Scenario\;2}=266\;\mu \text{Mol}/m^{2}/s.$$

If *T*_*pulse*_ is much longer, say 50 ms, Eq. (4) yields:

(9) $${\left\langle n\right\rangle }_{Scenario\;1}=30\;photons/pulse\;,{\left\langle n\right\rangle }_{Scenario\;2}=31\;photons/pulse.$$

For a duty cycle of 0.1 (*T*_*pulse*_ = 50 ms, *T*_*dark*_ = 450 ms), Eq. (6) yields:

(10) $$I_{av,cycle,Scenario\;1}=100\;\mu {\text{Mol}/m}^{2}/s\;,I_{av,cycle,Scenario\;2}=103\;\mu {\text{Mol}/m}^{2}/s$$

Scenario 1 always yields a cycle-averaged photon flux of 100 *μ*Mol/m^2^/s (the integrated flux, Eq. (1)). The result in Scenario 2 is appreciably larger than the integrated flux and approaches it when *T*_*pulse*_ is short (more significantly, <*n*> is small).

The message from the examples presented above is that, depending on the characteristics of the first-photon arrival time statistics, for small *T*_*pulse*_ (more significantly, small <*n*>), the difference between the true cycle-averaged photon flux and the integrated flux of Eq. (1) may be sizeable. The shorter *T*_*pulse*_ is, the more dramatic is the effect. The difference diminishes for sufficiently long *T*_*pulse*_ (more significantly, large <*n*>).

Let us now address the issue of the statistics of the arrival time of the first-photon, which may be absorbed during a pulse. Considering the enormous number of photo-systems within a cell and in a culture, one may safely assume that the starting time of a pulse occurs with a uniform probability distribution over the time interval, *Δt*, between the last photon, not absorbed during the pulse, and the next photon, which may be the first to be absorbed during the pulse. This choice is adopted in the following. The analysis then yields:

(11) $$\left\langle n\right\rangle =1/2+\left(T_{pulse}/\tau \right)+\left(T_{pulse}/\tau \right)\left(\left(T_{pulse}/2\;\tau \right)+1\right)\;\Gamma \left(0,\left(T_{pulse}/\tau \right)\right)-1/2\;e^{-\left(T_{pulse}/\tau \right)}\;\left(\left(T_{pulse}/\tau \right)+1\right)\;\underset{\left(T_{pulse}/\tau \right)\gg 1}{\to }1/2+\left(T_{pulse}/\tau \right)$$

(*τ* is defined in Eq. (3)) In Eq. (11), *Γ*(0, *x*) is the Incomplete Gamma function (Gradshtein and Ryzhik, 2007). The terms in the second and third lines of Eq. (11) fall off exponentially, so that, for large (*T*_*pulse*_/*τ*), the simplified, limiting expression holds.

Consider three examples for *I*0 = 1,000*μ*Mol/m^2^/s, *A* = 1nm^2^ and a duty cycle of 0.1. The traditional “integrated” value for *I*_*av*_ is 100*μ*Mol/m^2^/s. With Eqs. (5) and (11), the results are different. For, *T*_*pulse*_ = 1 ms, the result is *I*_*av*_ = 169*μ*Mol/m^2^/s, significantly higher than the 100*μ*Mol/m^2^/s integrated value of Eq. (1). For *T*_*pulse*_ = 0.1 ms the situation is much worse: *I*_*av*_ = 338*μ*Mol/m^2^/s. On the other hand, if the pulse length is 50 ms, *I*_*av*_ = 102*μ*Mol/m^2^/s.

## 3 Effect of photon-arrival time statistics: numerical demonstration

The results shown in the following were obtained by a numerical simulation. The flow-chart of the calculation is presented in the Appendix. The Mathematica program is available upon request.

**Figure 1:**
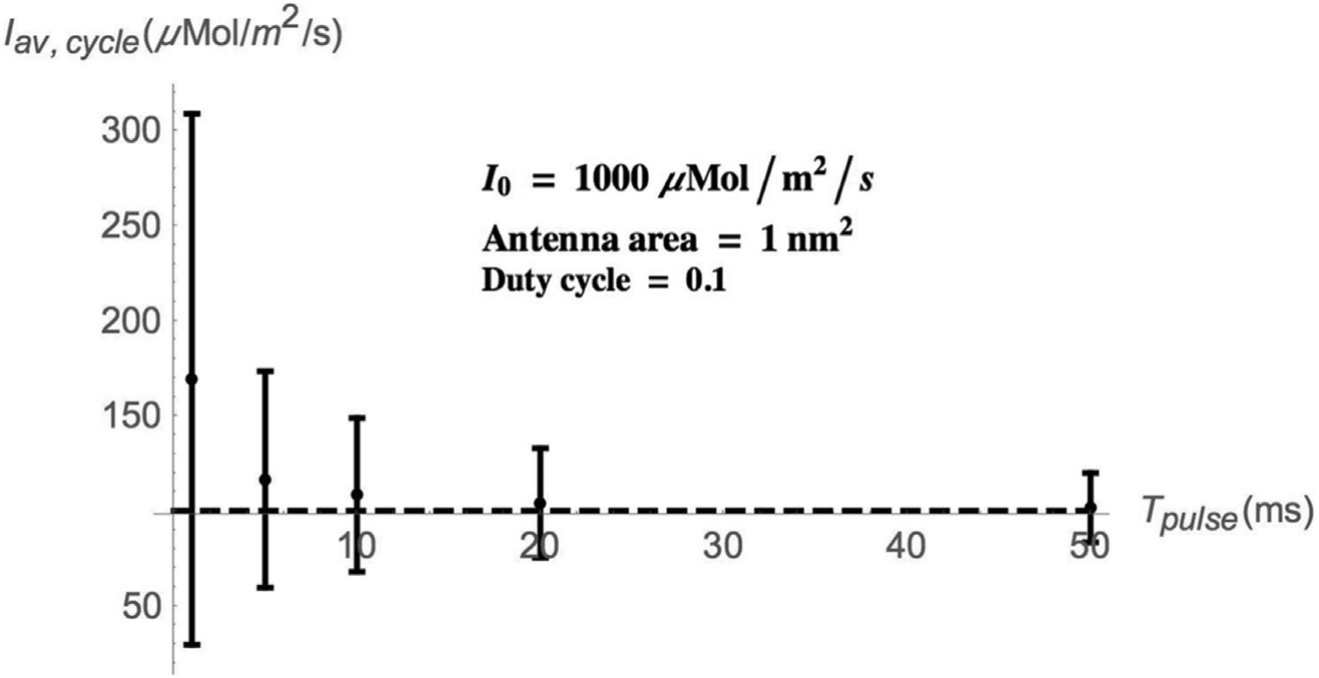
Cycle-averaged photon flux vs. pulse length.

Figure 1 shows the cycle-averaged photon flux vs. pulse time obtained when the statistics of random time gaps between consecutive photons are determined by Eq. (2) and first-photon arrival time statistics are taken into account. The standard deviations are obtained by the numerical simulation program.

For very short *T*_*pulse*_ (more important, small <*n*>), *I*_*av, cycle*_ significantly exceeds the integrated value of Eq. (1) (100 *μ*Mol/m^2^/s) and the statistical fluctuations are sizeable.

**Figure 2:**
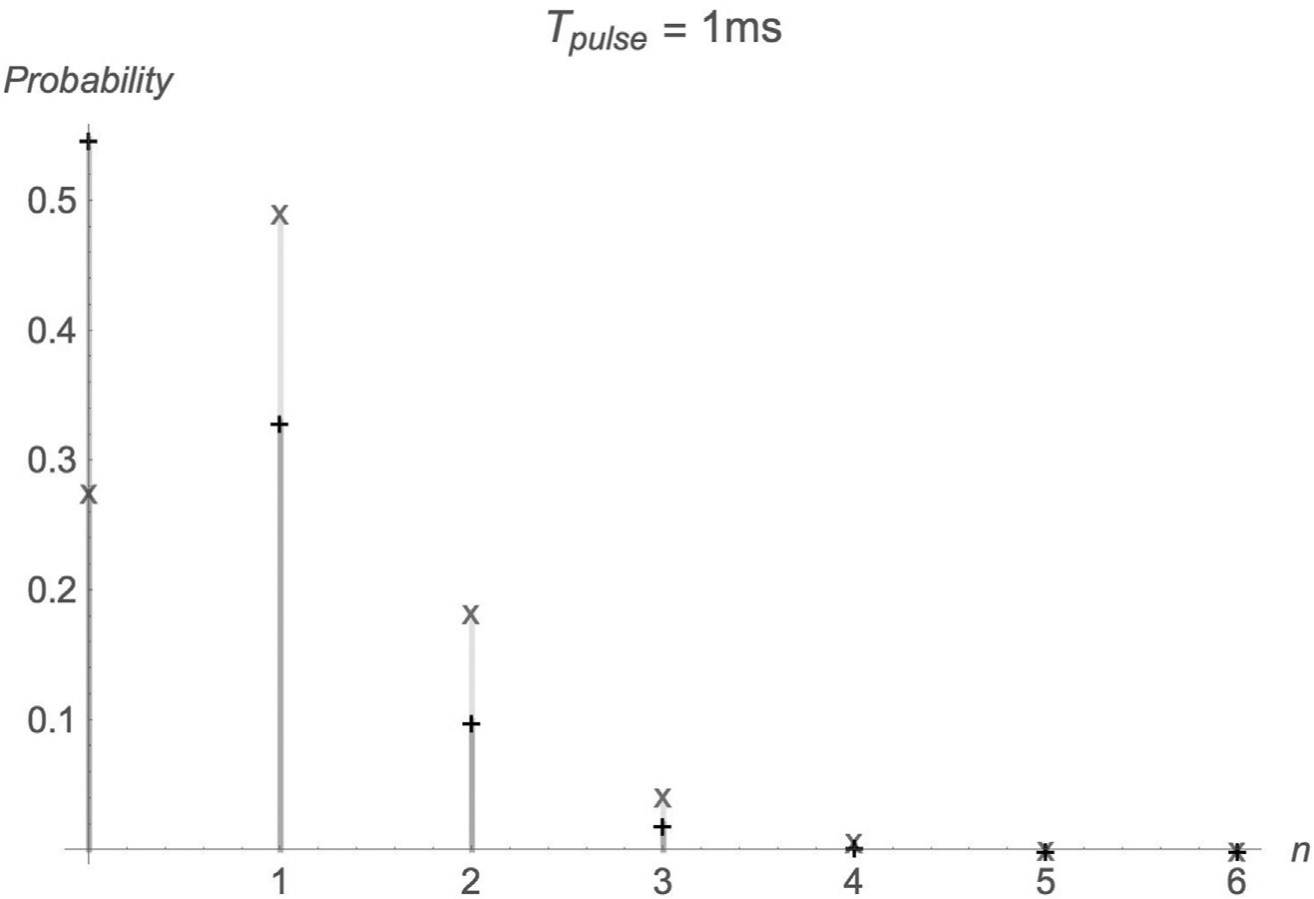
Probability distribution of absorbed photons number. Parameters as in Figure 1. x: 1^st^ photon arrival time statistics accounted for. +: 1^st^ photon arrival time statistics not accounted for.

**Figure 3:**
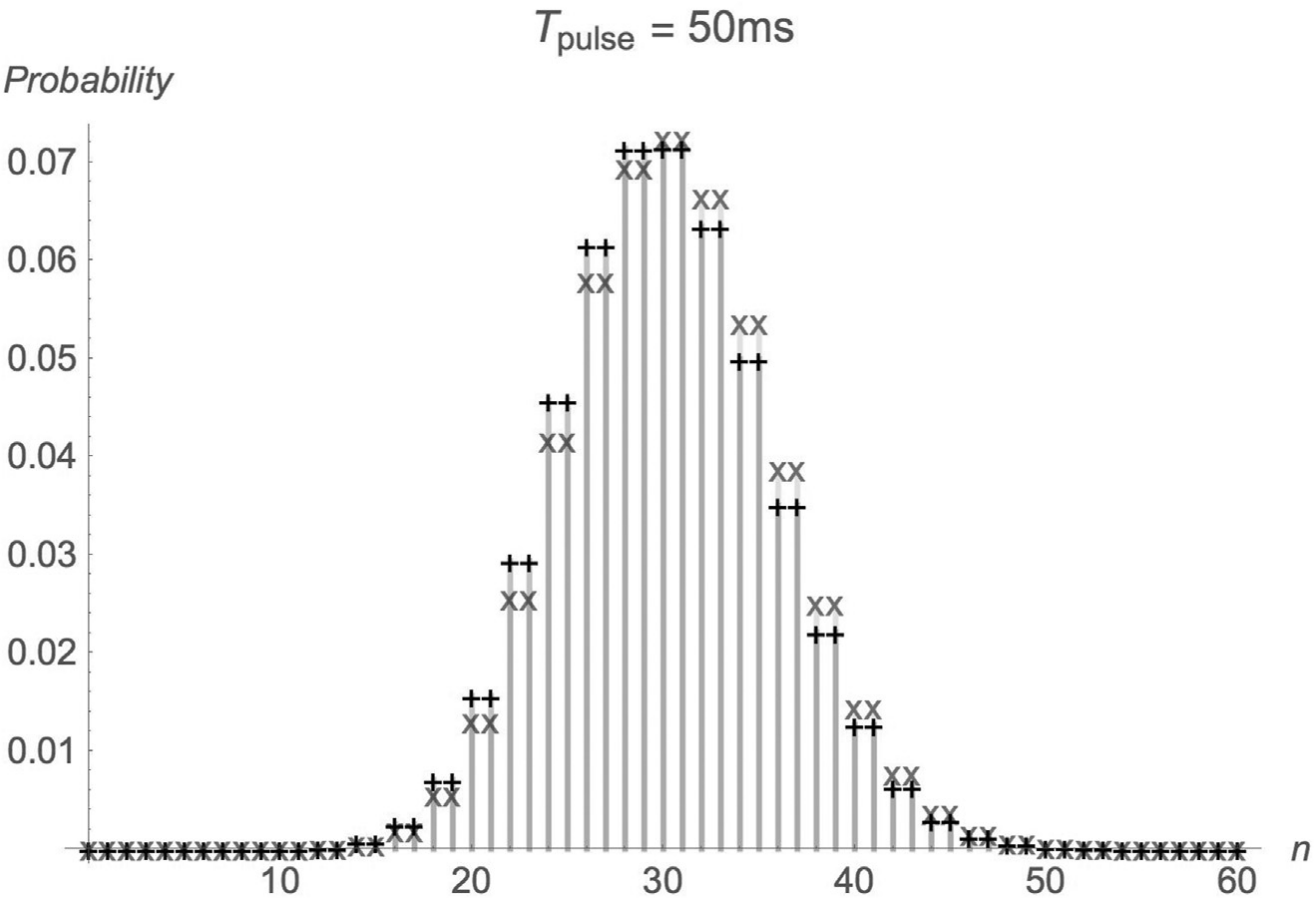
Probability distribution of absorbed photons number. Parameters as in Figures 1 and 2.

Figures 2 and 3 show the probability distributions of absorbed photons in two scenarios: First-photon arrival time statistics accounted for and is not accounted for. Parameters are as in Figure 1. It is evident that, when the pulse duration is short (more importantly, <*n*>, the average number of photons absorbed during a pulse is small), the difference between the results, in which the first-photon arrival time statistics are accounted for, and those, in which they are not accounted for, is significant. As the pulse time becomes sufficiently long (more importantly, <*n*> is large), the difference between the two calculations diminishes.

## 4 Concluding comments

The foregoing discussion indicates that, when <*n*>, the average number of photons absorbed during a pulse is small, a comparison of biomass production rates under a pulsed-light regime with the rates under continuous illumination at the integrated intensity of Eq. (1) may be misleading. Furthermore, even use of the correct cycle averaged photon flux (Eq. (11)) does not convey the full picture. The reason is that, in a pulsed-light regime, the standard deviation around the correct average photon flux may be so high, that further statistical analysis is required.

## Declarations

### Author contribution statement

Yair Zarmi, Ph.D: Conceived and designed the experiments; Performed the experiments; Analyzed and interpreted the data; Contributed reagents, materials, analysis tools or data; Wrote the paper.

### Funding statement

This research did not receive any specific grant from funding agencies in the public, commercial, or not-for-profit sectors.

### Data availability statement

No data was used for the research described in the article.

### Declaration of interest’s statement

The authors declare no competing interests.

### Additional information

No additional information is available for this paper.

## Figures and Tables

**Figure:**